# Evans blue dye-enhanced imaging of the brain microvessels using spectral focusing coherent anti-Stokes Raman scattering microscopy

**DOI:** 10.1371/journal.pone.0185519

**Published:** 2017-10-19

**Authors:** Bo-Ram Lee, Kyung-Il Joo, Eun Sook Choi, Junghoon Jahng, Hyunmin Kim, Eunjoo Kim

**Affiliations:** 1 Companion Diagnostics and Medical Technology Research Group, DGIST, Daegu, Republic of Korea; 2 School of Electronics Engineering, Kyungpook National University, Daegu, Republic of Korea; 3 Department of Physics and Astronomy, University of California, Irvine, California, United States of America; Hungarian Academy of Sciences, HUNGARY

## Abstract

We performed dye-enhanced imaging of mouse brain microvessels using spectral focusing coherent anti-Stokes Raman scattering (SF-CARS) microscopy. The resonant signals from C-H stretching in forward CARS usually show high background intensity in tissues, which makes CARS imaging of microvessels difficult. In this study, epi-detection of back-scattered SF-CARS signals showed a negligible background, but the overall intensity of resonant CARS signals was too low to observe the network of brain microvessels. Therefore, Evans blue (EB) dye was used as contrasting agent to enhance the back-scattered SF-CARS signals. Breakdown of brain microvessels by inducing hemorrhage in a mouse was clearly visualized using backward SF-CARS signals, following intravenous injection of EB. The improved visualization of brain microvessels with EB enhanced the sensitivity of SF-CARS, detecting not only the blood vessels themselves but their integrity as well in the brain vasculature.

## Introduction

The imaging of microvessels in the animal brain is crucial for observing the integrity of the blood-brain barrier (BBB) in neurodegenerative diseases [1]. It has been shown that the integrity of the brain microvessels was damaged in certain conditions such as multiple sclerosis, Alzheimer’s disease, cerebral malaria, and hemorrhage by stroke or traumatic injury [2–6]. There are several methods available to visualize brain microvascular networks. To observe endothelial cell surface markers such as CD31, an intravenous infusion of fluorescent dye-labeled antibodies into the blood plasma is required [7,8]. However, the antibody-directed visualization of brain microvessels only provides an indirect estimate using the target protein distribution on brain endothelial cells. On the other hand, exogenous dyes such as sodium fluorescein and Evans blue dye (EB) could be injected intravenously for observation of blood vessels [9, 10]. In an intravenous system, exogenous dyes act as tracers, thus they contrast the blood vessels against the surrounding tissues.

Coherent anti-Stokes Raman scattering (CARS) is a nonlinear four-wave mixing (FWM) process used to enhance a weak Raman signal. In the CARS process, a pump laser beam interacts with a Stokes laser beam, producing an anti-Stokes signal. When the frequency difference between the pump and Stokes beams matches the frequency of a vibrational mode, such as the CH_2_, CH_3_, and O-H stretching at 2,800–3,100 cm^-1^, the molecular oscillators are coherently driven. Cell biology and tissue imaging have benefited from CARS microscopy because it enables chemically specific, label-free imaging at the sub-micron scale [11]. To date, CARS microscopy has been used for high-resolution imaging of label-free lipid droplets, myelination of neurons, cell proliferation in bioengineered tissue scaffolds, and lipid distribution in tissue structures [12,13].

It is feasible to image brain microvessels with CARS microscopy because they are composed of lipid and protein-rich endothelial cells. However, the integrity of the vascular network in the brain cannot be successfully observed by CARS microscopy because the background signals from the surrounding tissue are usually strong, thereby making it difficult to obtain clear network images of the brain microvessels [14, 15]. The interaction of the same vibration frequencies with the molecular electronic levels can generate a non-resonant FWM signal spectrally distinguishable from the resonant CARS. The non-resonant background can limit the sensitivity and alter the CARS spectra as compared with spontaneous Raman spectra. Therefore, the development of other detection schemes for CARS microscopy is necessary to visualize the vasculature specifically and overcome the non-resonant background signals.

As a technique for achieving chemically selective imaging with reduced non-resonant background contributions, spectral-focusing CARS (SF-CARS) with a femtosecond laser system is presented [16–18]. The idea of using a chirped femtosecond pulse for coherent Raman scattering imaging (hyperspectral or spectrally focused coherent Raman imaging) modality has been recently developed to visualize the chemically sensitive signals without requiring a change of the colors of one of the excitation photons. That is, in this system, the Raman-like spectral profiles are resolved by simply changing the position of the time overlap between two stretched pump and Stokes beams in a spectrometer-free fashion.

In more detail, a linearly chirped and stretched (~1 ps) pump pulse generates a CARS signal only when overlapped in time with the Stokes pulse, offering spectral information according to the extent of the pump/probe time delay. As another approach to reduce the non-resonant background, researchers reported that if a tissue sample is detected in the epi-detection, non-resonant signals from tissue components as well as the solvent are almost completely eliminated [19]. Such epi-detection is possible when the sample is highly scattering.

In this study, by using a home-built SF-CARS microscopic system, 200 μm-thick brain tissue samples from mice were studied in an aqueous environment. Because these tissue samples were expected to have significantly enhancing forward CARS signals due to the opaque light scattering condition, epi-detection was used in the visualization of the microvessels to separate them from the non-resonant background signal. To this end, a highly polarizable EB dye [20] was employed to enhance the microvessel-specific CARS signals to supplement the decreased signal intensity in the course of epi-detection of back-scattered SF-CARS signals, following intravenous injection of EB into mice. The Kramers-Kronig phase-retrieval method was also adopted to analyze the SF-CARS spectra and remove non-resonant background signals [21]. Here, we propose a customized condition for preparing tissue samples to observe brain microvessels by using SF-CARS microscopy.

## Materials and methods

### Spectral focusing CARS microscopy setup, measurement, and data analysis

A home-built coherent anti-Stokes Raman scattering (CARS) microscopy system was employed to visualize brain vascular structures in this study (S1 Fig). The CARS contrast targeting a 2,956 cm^-1^ C-H vibrational mode was obtained by combining 1,041 nm Stokes and 796 nm pump pulse trains emitted from a dual beam mode-locked erbium-doped fiber laser (Insight deepsee dual, Spectra-physics). Spatial collinear alignment of the two beams was achieved by a dichroic mirror (DMSP1000R, Thorlabs) while the temporal overlap was created with a motorized translation stage (SGSP46-500, Sigma-Koki). Spectral focusing (~1 ps, ~3 nm) was achieved by passing these beams (120 fs for pump; 220 fs for Stokes, 80 MHz) through two 12-cm long SF 57 glasses [22]. A commercially-available galvanometric scanning system (FluoView 1000, Olympus) implemented with an inverted microscope (IX83, Olympus) was employed to allow 3D image scanning of the sample after passing through a 1.35 NA objective lens (UPlanFLN, Olympus). To achieve minimal sample damage, the pulse powers at the sample were managed by less than the level of ~ 5 mW and ~ 35 mW for the pump and Stokes beams, respectively, using half-wave plates (ACWP-700-1000 for pump; ACWP-1000-1600 for Stokes, CVI) and Glen-Thomson polarizers (PTOL-10.0-670-1064, CVI). The CARS signal was collected by a photomultiplier tube (R3896 PMT, Hamamatsu) after passing a condenser (NA = 0.55) and a 641 nm BF3 bandpass filter (FF02-641/75, Semrock).

For SF-CARS spectra, the SF-CARS images were collected as a function of inter-pulse time delay from ~2,750 cm^-1^ to ~3,150 cm^-1^ using a center wavelength of 796 nm for the pump and a 1,041 nm wavelength for the Stokes pulses (center wavenumber as ~2,956 cm^-1^). Interferometric measurements (AA-M, Avesta Inc) revealed the degrees of stretching for the pump and Stokes pulses with ~1.3 ps and ~0.8 ps, respectively. In this work, the linear chirping factor ($\beta =\sqrt{{\left(\pi c\Delta \lambda /\lambda^{2}\Delta \tau \right)}^{2}-4{\left(ln2\right)}^{2}/\Delta \tau^{4}}$, where c, *λ*, *Δλ*, and *Δτ* are the speed of light, pulse wavelength, chirped pulse bandwidth, and chirped pulse duration, respectively, of the pump and Stokes beams were calculated to be ~3.3 × 10^24^ s^-2^ and 2.4 × 10^24^ s^-2^, respectively. The non-resonant SF-CARS spectra were simultaneously constructed from each SF-CARS image of the glass background to eliminate the non-resonant term and Gaussian-like overlapping factor of $\text{exp}{\left(-\frac{{\text{t}}_{0}}{2\tau }\right)}^{2}$ (t_0_: pulse delay; τ: pulse duration time), revealing the full-width at half maximum (FWHM) of the overlap of the pump and Stokes beams at ~26 cm^-1^. A comparison with the Raman scattering signal of dimethylsulfoxide solution (S2 Fig) revealed the peak positions of the SF-CARS signal to be well-coordinated with the vibrational modes of the spontaneous Raman spectrum. Additionally, the linearity of the chirping was calibrated (inset graph) to ~0.47 cm^-1^/μm, showing a close match to the literature (~0.5 cm^-1^/μm) for a similar length (25.4 cm) SF57 glass rod [22]. It took approximately ~2.5 min (1 s for image taking plus 0.5 s for the stage transition over 100 SF-CARS image sets) to obtain one SF-CARS spectrum. The post-imaging data processing was performed using a phase-retrieval method based on a modified Kramers-Kronig transformation (KK-transform) combined with a phase/amplitude considering error correction algorithm, as reported in the literature [23]. We modified the demo version of the phase-retrieval code written in Matlab and applied it to the SF-CARS dataset to achieve the final form of the phase-corrected spectra.

### Animals

Male C57BL6 mice were purchased from Hyo-Chang Science (Daegu, Korea) and used at 6–8 weeks of age. On arrival, the mice were randomly assigned to experimental groups, followed by an acclimation period of at least 3 days before initiating the experiments. Four mice were housed per cage, and the cages were maintained in a 12-h light/dark cycle with food and water *ad libitum*. The animals were maintained at DGIST Animal Laboratory (Daegu, Korea) in accordance with the Institutional Animal Care Guidelines. The Animal Care and Use Committee of DGIST approved all animal protocols.

### Sample preparation

For all samples, the harvested brain tissue was fixed overnight in 4% paraformaldehyde (PFA) in phosphate buffered saline (PBS) at 4°C. Tissue sections of 200 μm were cut using a vibrotome (Leica VT1200 S, Wetzlar).

The samples were as follows:

*(BC-)/(EB-)* This sample was free from both blood cells (BC) and Evans Blue (EB), i.e. it can be regarded as the absolute control. It was prepared by perfusing the animals with 4% PFA immediately after sacrifice to wash out all blood cells from the blood vessels.
*(BC+)/(EB-)* These samples contained blood cells. There was no perfusion after sacrifice.
*(BC+)/(EB+)* These samples contained both blood cells and Evans Blue. There was no perfusion after sacrifice; EB was injected (1% in PBS, 100 μl) intravenously 4 h before sacrificing the animal.
*(BC-)/(EB+)* These samples contained EB dye. It was prepared by perfusing the animals with PBS to wash out all blood vessels, immediately followed by injection of 1% EB solution through the aorta. Cerebral hemorrhage was induced by the stereotactic injection of collagenase (0.5 U in 2 μL PBS) into the striatum using a stereotaxic micromanipulator (David Kopf Instruments). After 0 h, 3 h, and 24 h, mice were sacrificed without perfusion, and 1% EB (100 μL) was injected intravenously 4 h before sacrifice for each group. The tissue slices were prepared at 200 μm thickness, as described above.

## Results and discussion

### SF-CARS imaging and signal origin configuration

**Fig 1:**
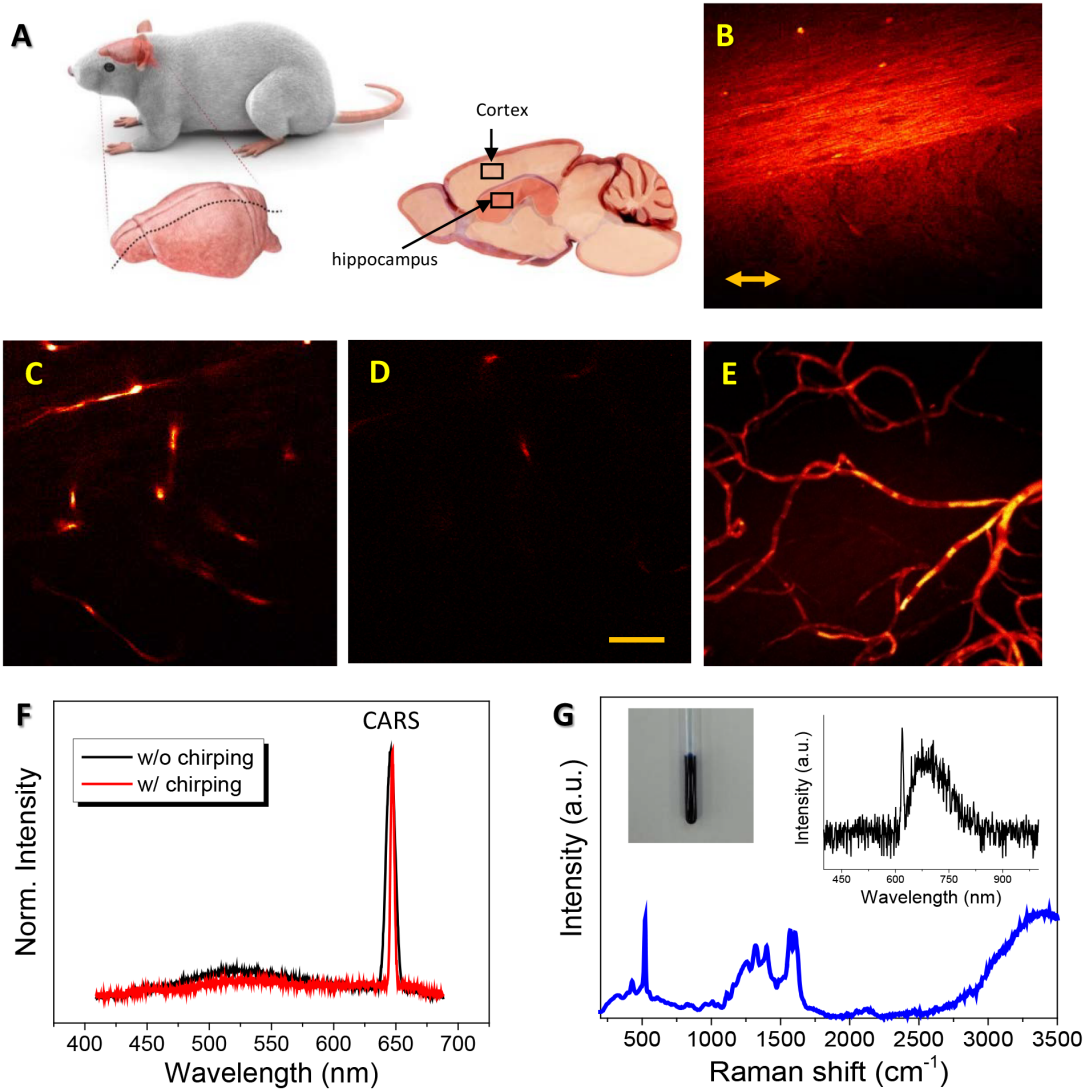
Preparation of mouse brain tissues and instrument. (A) An illustrative image shows tissue sections (200 μm thickness) used in this work (cerebral cortex and hippocampus). (B-D) Backscattered spectral focusing coherent anti-Stokes Raman scattering (SF-CARS) images (tuned to 2,920 cm^-1^) taken from the blood vessel of a (BC+)/(EB+) sample containing blood cells and Evans blue, located in cortex tissue at the depth of (B) 10 μm, (C) 100 μm, and (D) 150 μm tuned to 2920 cm^-1^. (E) The image created by stacking ~100 SF-CARS images in the z-direction. The scale bar indicates 20 μm. The double-sided arrow in (B) indicates the direction of the polarization of the two parallel beams (pump and Stokes). The polarization direction is identical for all following images. (F) The spectra obtained from (BC+)/(EB+) by 5 mW 796 nm pump and 35 mW 1041 nm Stokes beams in the presence and absence of beam chirping is compared with that of EB dye solution. (G) Raman spectra for EB dye powder. Upper left inset is a photograph of EB dye contained in the NMR tube. The upper right inset is the fluorescent spectra with the excitation wavelength at 620 nm.

Fig 1A schematically shows the illustrative anatomy of a specimen (cerebral cortex and hippocampus) used in this study. Fig 1B–1D shows the cross-sectional views taken by the backscattered SF-CARS imaging technique in the presence of blood cells and EB dye (BC+)/(EB+) for different depths from the cut plane at 10 μm (B), 100 μm (C), and 150 μm (D). A very large river-like structure was observed in a transverse plane near the surface, while small-sized vascular structures appeared in and out as the scanning position went deeper, which likely became connected vessels as observed on a z-directionally merged image. The large river-like structure seems to be either nerve fibers including axons and myelin sheaths, or collagen fibers, or both. Fig 1E is the image from a 200-μm thick tissue section created by z-stacking of SF-CARS images acquired every 1 μm. This final image shows clear blood vessels and their integrity in the brain vasculature. To observe the Raman shift around 2,800–3,100 cm^-1^, which was attributed to CH_2_ or CH_3_ stretching in the lipids and proteins of the brain vasculature, we adjusted the delay between the 5-mW 796-nm pump and 35-mW 1041-nm Stokes beam to enhance the amplitude of the signal around 644 nm.

Fig 1F shows the SF-CARS signal from backward scattering clearly observed at 644 nm from the brain tissue prepared without perfusion of the mice (BC+)/(EB+). It was measured with and without chirping of the beams, and the peak at 644 nm with chirping showed a narrower (~3 nm) band compared to that without chirping (~7 nm). Raman spectra obtained for EB powder showed that EB dye did not exhibit clear resonant Raman signals by CH_2_ or CH_3_ stretching between 2,800–3,100 cm^-1^ (Fig 1G). These results indicate that the SF-CARS signal of the EB-containing tissue (Fig 1F) could not originate solely from the vibrational signal of EB itself distributed in the brain microvessels, since EB does not contain a C-H-rich chain (S3 Fig). The inset of Fig 1G shows the EB fluorescence spectrum excited at 620 nm, exhibiting its maximum emission around 680 nm.

In the other hand, the CARS signal in Fig 1F could have originated from microvessels and BCs, which were composed of CH_2_ and CH_3_-rich protein and lipid molecules. Allegedly, the biological source for stimulated Raman scattering (SRS) and CARS of blood vessels is red blood cells (RBCs), which are the most abundant cells in the whole blood [24]. In RBCs, the heme compound in hemoglobin protein is fluorescent, and it was expected to contribute to resonant Raman signals of blood vessels, which was supported by the Raman and FTIR transmittance intensity of hemin (heme compound with a chloride ligand), as shown in S4 Fig. Therefore, the SF-CARS signal in Fig 1F could include the reflected forward-scattered resonant signal of BCs and backscattered resonant hemin in blood vessels.

**Fig 2:**
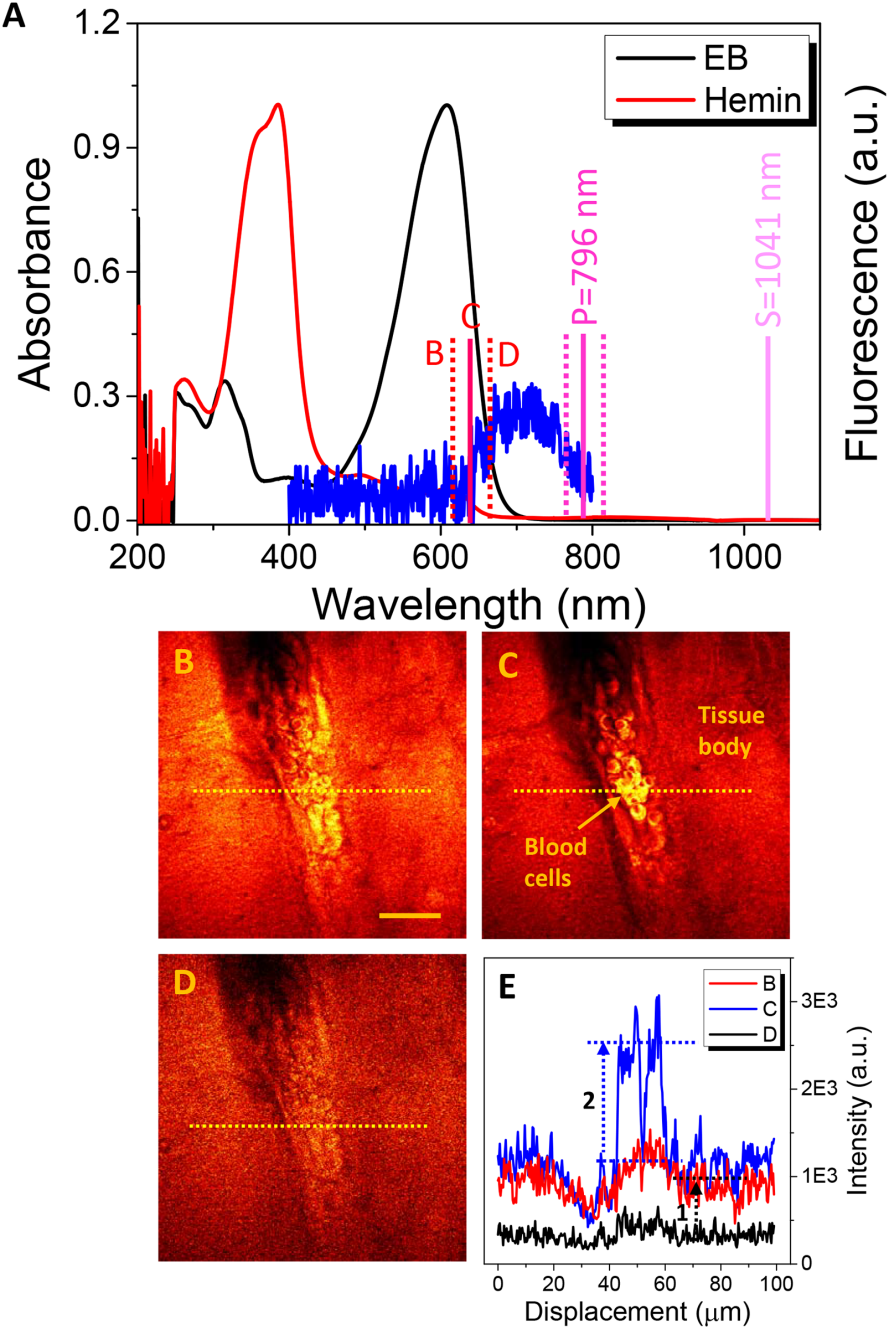
Optical analysis of hemin and Evans blue. (A) Absorbance spectra for EB (black) and hemin (red); fluorescent spectrum of EB with excitation at 620 nm (blue). The forward SF-CARS images of the brain vascular structure (BC+)/(EB+) when the wavelength of the pump beam is tuned to (B) 3214 cm^-1^ (λ_CARS_ = 623 nm) (C) 2957 cm^-1^ (λ_CARS_ = 644 nm,) and (D) 2740 cm^-1^ (λ_CARS_ = 662 nm,). (E) The intensity line profile over the dotted lines in (B), (C), and (D). The scale bar indicates 30 μm.

Fig 2A shows the difference of EB and hemin as contrasting agents in observation of brain microvessels by SF-CARS. The optical absorption of the hemin (λ_max_ = 388 nm) was far from the linear/nonlinear excitation band (644 nm) from the pump (796 nm) and Stokes beams (1041 nm) used in this study. On the other hand, the optical absorption of EB dye (λ_max_ = 620 nm) was partially overlapped with the electronic absorption as much as 644 nm, which was expected to cause the amplification of CARS signals from blood vessels by the existence of EB in blood circulation. The electronic absorption property of EB near 644 nm might exert a role in backscattered detection of SF-CARS, as a non-resonant signal from blood vessels.

In Fig 2B–2D, the forward SF-CARS signals of (BC+)/(EB+) samples are shown. For a clearer understanding of the origin of forward CARS signals, we zoomed into the 2,800–3,100 cm^-1^ C-H stretching region. The difference between B and D (1 in Fig 2E) originates from the tailed resonant signal from water at 3,214 cm^-1^, while the difference between C and B is due to resonant C-H vibration from the blood cells. Also, the intensity increase (2) in Fig 2E was due to the combined contribution of the resonant term of the CARS from blood cells (C-H-rich cellular components) and some portion of the resonant/non-resonant CARS from the EB dye (Figs 1G and 2A). It has been reported that the EB structure could also have strong non-resonant optical properties for use as a contrast agent for photoacoustic imaging [25,26].

### Contrast effect of Evans blue in SF-CARS imaging

**Fig 3:**
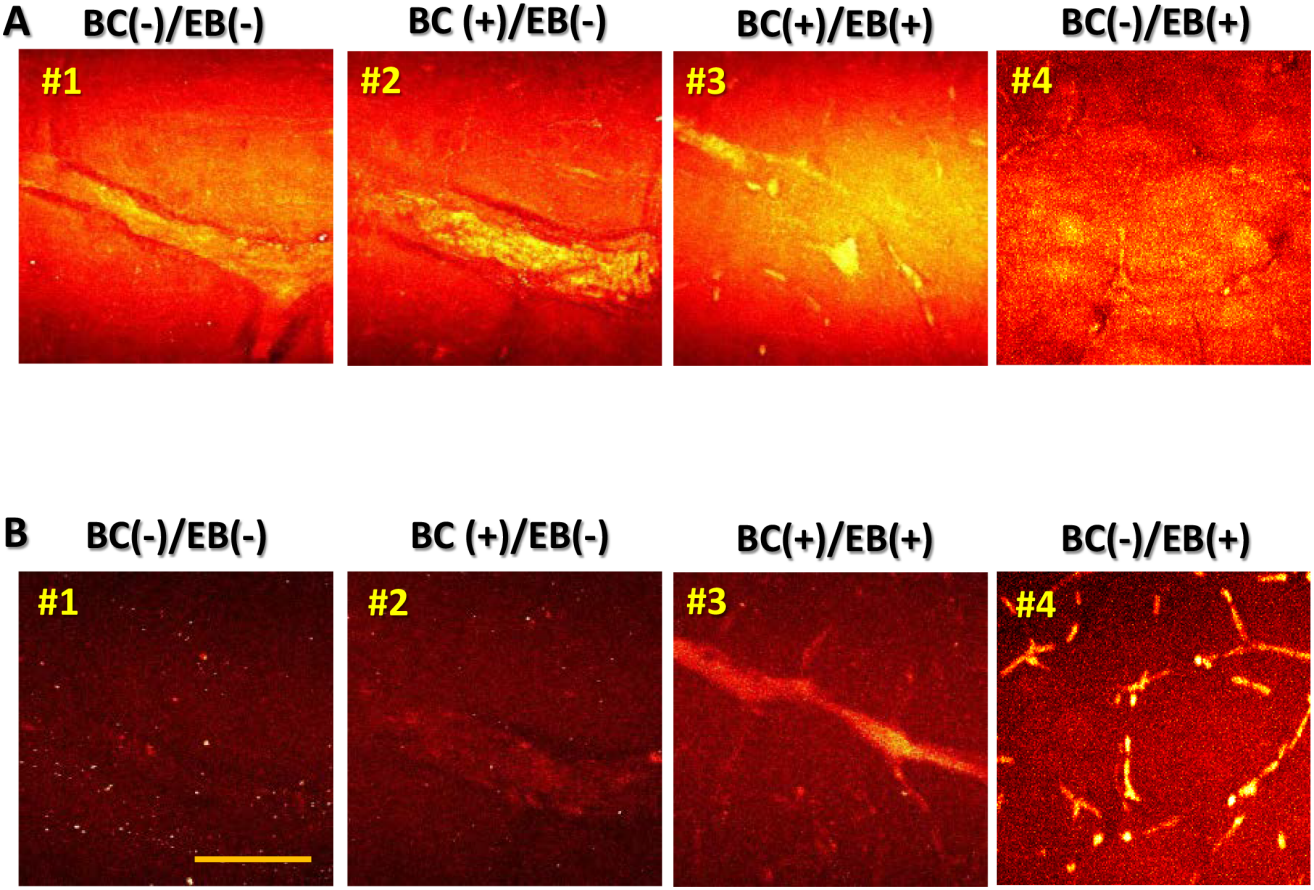
Observation of brain microvessels using SF-CARS. Merges of 100×1 μm stacked SF-CARS images captured from the brain cortex (200 μm thickness) by forward (A) and backward (B) detection at 2,920 cm^-1^. The scale bar indicates 50 μm.

In Fig 3A, the blood vessels and the surrounding tissue in the brain sections for each group are visualized in bright images produced by the forward signal of SF-CARS at 2,920 cm^-1^. In the present study, four types of samples were prepared with or without perfusion of blood vessels and injection of EB: (BC-)/(EB-), (BC+)/(EB-), (BC+)/(EB+), and (BC-)/(EB+).

The images in Fig 3 were re-constructed by stacking ~100 SF-CARS images separated by 1 μm each in the z-direction. Because brain tissue is a complex matrix composed of C-H-rich biomolecules such as proteins and lipids, strong background signals were visualized irrespective of the presence or absence of either BCs or EB in the microvessels. In addition, the SF-CARS signal of water could be simultaneously detected in the forward direction, which was previously reported to cause high non-resonant background signals [27]. Although the sensitivity of the forward CARS detection is usually background-limited, the epi-detection of back-scattered SF-CARS signals showed improved imaging resolution due to the reduced non-resonant background [28]. If the back-scattered SF-CARS signal from water was almost zero, it greatly improved the sensitivity. As shown in Fig 3B, the background signal in back-scattered SF-CARS was negligible. However, the blood vessels in (BC-)/(EB-) and (BC+)/(EB-) disappeared from the images obtained by epi-detection. The (BC+)/(EB+) and (BC-)/(EB+) wetted tissue samples exhibited contrasting signals from the blood vessels due to the reduction of the high non-resonant background from forward-directional signals.

Because EB did not show a strong resonant Raman signal at 2,800–3,100 cm^-1^ (Fig 1G and S3 Fig), EB dye could not be used as a contrasting agent for forward scattering of blood vessels. Because the non-resonant signal from small objects is retained in backscattered detection of CARS, the electronic non-resonant signal of EB itself would not be reduced in the epi-detection [29]. Therefore, resonant/non-resonant signals of EB could contrast the blood vessels without BCs in backscattered detection of SF-CARS.

### Spectral analysis of the brain microvessels by SF-CARS microscopy

**Fig 4:**
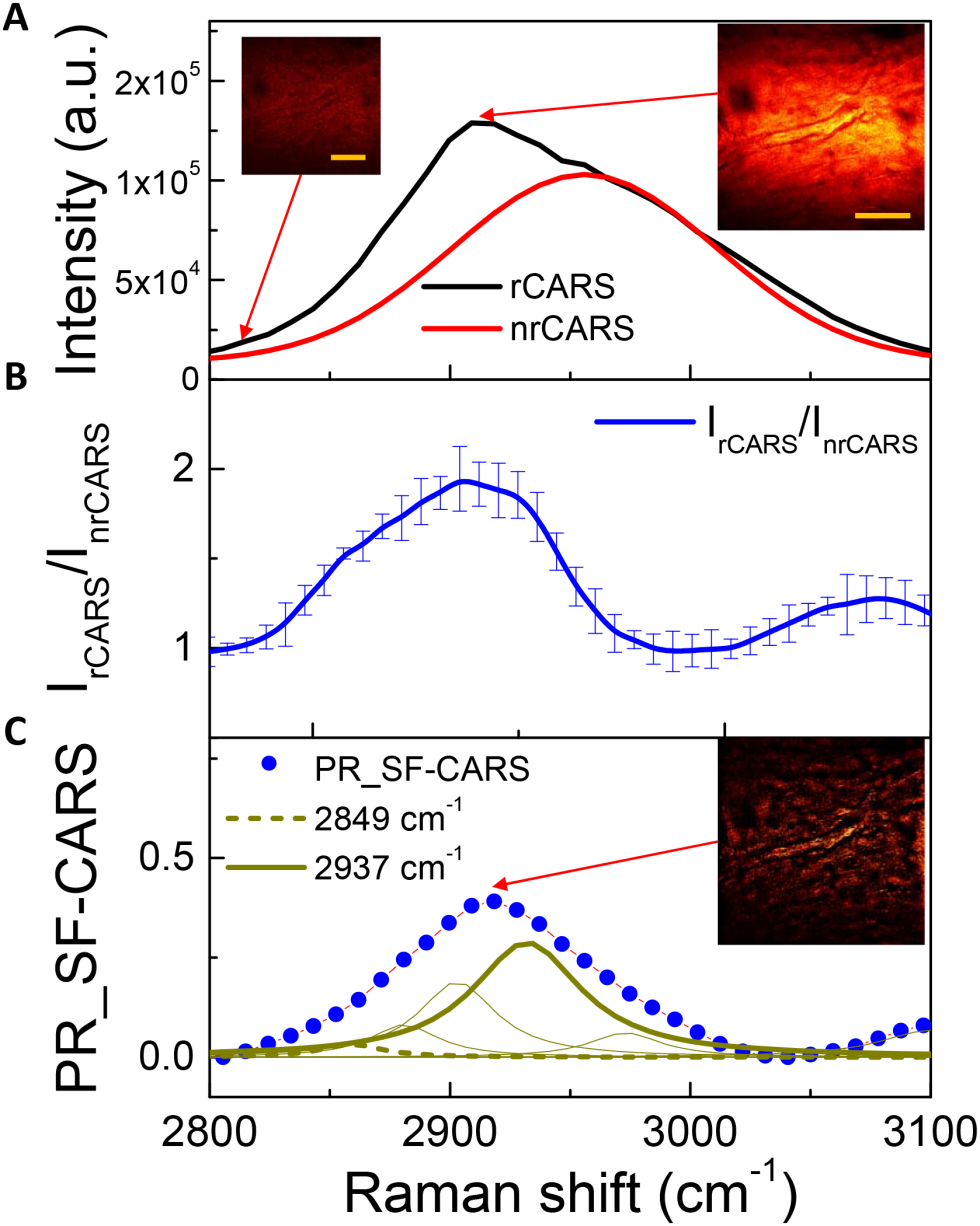
Forward phase retrieved SF-CARS spectra for brain microvessels. (A) The SF-CARS spectra from the sample (rCARS; black) and backgrounds (nrCARS; red. Left-side inset image was taken at 2,820 cm^-1^ while the right-side images were taken at 2,920 cm^-1^. The scale bars indicate 20 μm. (B) Intensity ratio between rCARS and nrCARS. (C) Decomposition of the phase-retrieved SF-CARS spectra created by the modified Kramers-Kronig transformation method.

CARS microscopic studies have mostly relied on the strong CH_2_ and CH_3_ stretching vibrations around 2,800–3,100 cm^-1^ by the lipids and proteins in tissues [30,31]. Fig 4A shows representative, overlaid spectra of forward SF-CARS signals from vascular structures and the glass background; Fig 4B is the SF-CARS spectrum by taking the relative ratio between a resonant and a non-resonant signal (I_hCARS_/I_nrCARS_) from the brain microvessels at middle depth of the tissue section. Also, a phase-retrieved SF-CARS (PR-SF-CARS) spectrum by KK-transform is given to remove possible errors during the SF-CARS acquisition process in Fig 4C with multiple peak deconvolution [32] for more analytical information from the vibrationally congested region. Note that the relative portions of 2,849 cm^-1^ and 2,937 cm^-1^ in the deconvoluted peaks were 5% and 35%, respectively, regardless of a small variance within 2%. A broad peak centered at 3,100 cm^-1^ was also observed with a lower peak intensity, which corresponded to the tail of the resonant mode of water. A general spectral trend observed herein is similar to that reported for tissues in the literature [33]. Here, it should be noted that the 4% PFA fixation of the specimen did not significantly contaminate CARS contrast [34]. Also, the excellence of the background removal effect in the PR-SF-CARS image was noticeable, as suggested in the inset of Fig 4C, compared to the inset SF-CARS image in Fig 4A.

**Fig 5:**
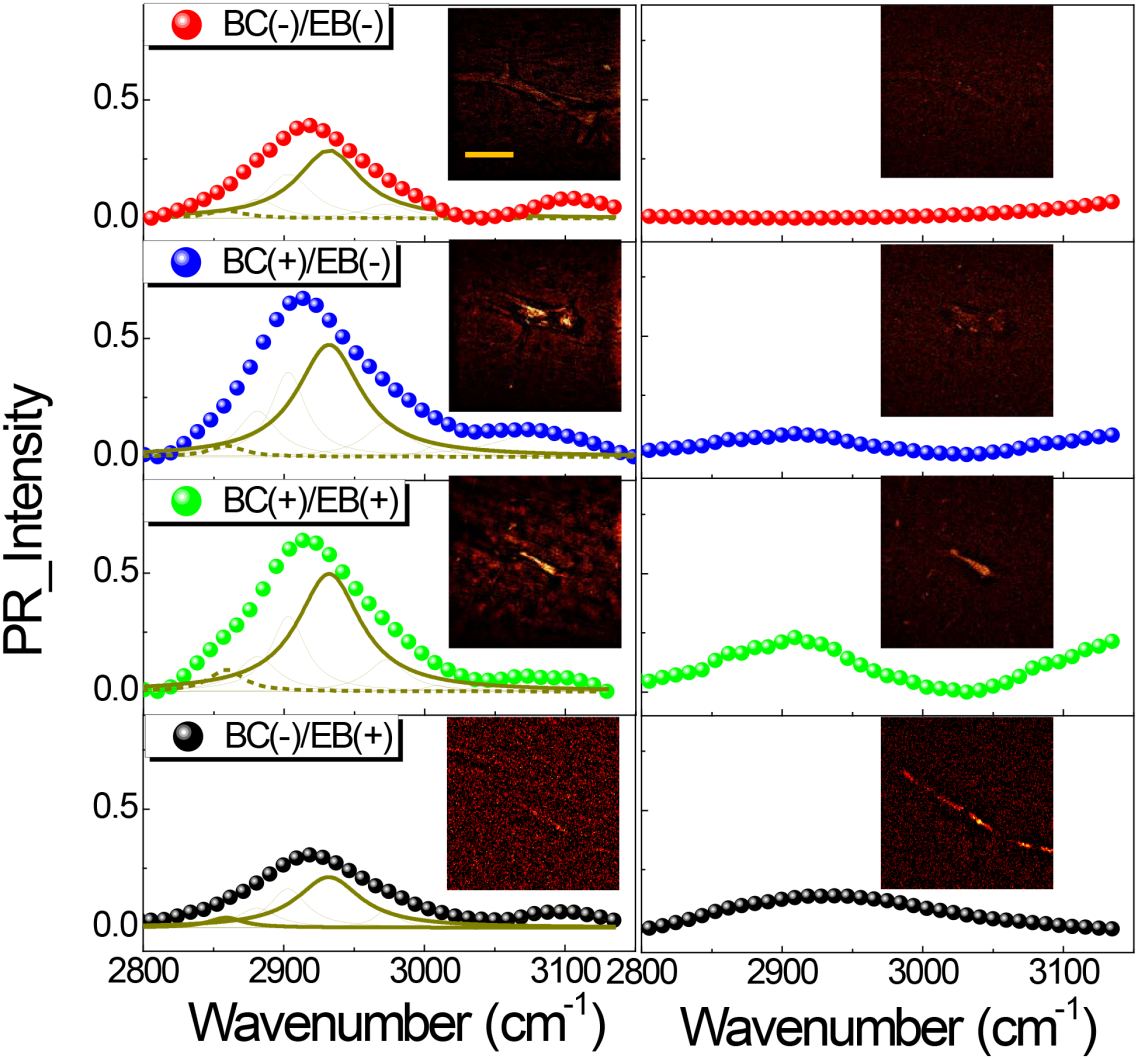
Comparison of forward and backward SF-CARS on contrasting agent effects. Forward (left) and backward (right) phase-retrieved SF-CARS spectra of brain microvessels. Inset images are the representative forward (left) and backward (right) SF-CARS images taken near the peak (~ 2920 cm^-1^) region. The scale bar indicates 10 μm.

As summarized in the left panel of Fig 5, the forward SF-CARS signals by C-H stretching from the vasculature were greater in the BC(+) samples ((BC+)/(EB-) and (BC+)/(EB+)) than in the BC(-) samples. This result indicated that brain microvessels with blood cells led to an enhanced intensity of resonant signals compared to those of EB-containing vasculatures (see also Fig 2C). EB dye in the blood stream was not related to the changes in resonant intensity from the vasculature, which coincided with the EB dye spectrum without a strong peak around 2,850–2,950 cm^-1^, as shown in Fig 1G. Taken together, the increased intensity of the resonant Raman shift by forward scattering originated from blood cells, and the presence of EB dye did not correlate with this intensification of the vibrational Raman signal.

The right panel in Fig 5 shows the spectra obtained from the brain microvessels by epi-detection of back-scattered SF-CARS. The backscattered signal observed at 2,800–3,100 cm^-1^ was noticeable in the (EB+) tissues ((BC+)/(EB+) and (BC-)/(EB+)), and there was no signal from the (BC-)/(EB-) and it was very low in the (BC+)/(EB-) sample. Even though the intensity of phase-retrieved CARS spectra was dramatically decreased in backward scattering, the vascular structure harboring EB was observed clearly by epi-detection. These results indicate that in the backward CARS the presence of the EB is the decisive factor in imaging of vasculatures by SF-CARS, which was attributed to the optical property of EB by emitting non-resonant signals from it.

### Tissue dependence of SF-CARS signals

**Fig 6:**
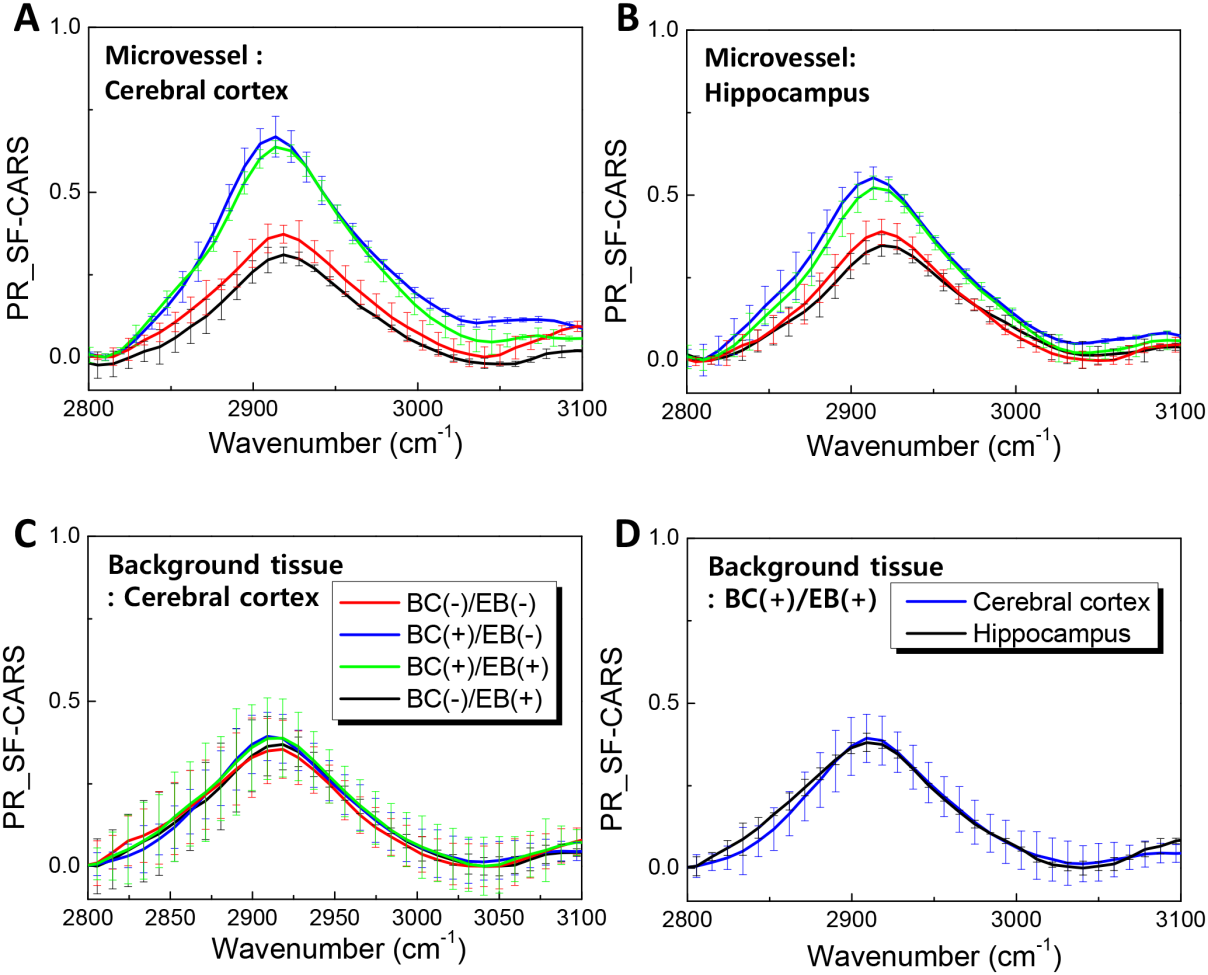
Tissue dependence of phase retrieved SF-CARS signals. (A-C) Forward SF-CARS spectra of microvessels in the cortex (A) and hippocampus (B), and background tissue of the cerebral cortex for each treatment (C). (D) SF-CARS spectra for the background tissue of the cerebral cortex and hippocampus.

Fig 6A and 6B show the forward-scattered PR-SF-CARS signals (at 2920 cm^-1^) for the vasculature in the cerebral cortex and hippocampus. The increased intensity of resonant C-H signals in BC-containing tissues as compared to those without BCs was commonly observed both in the cerebral cortex and hippocampal vessels. On the other hand, the C-H signals from the matrix tissue was not significantly different, irrespective of the localization of BCs or EB dye in blood vessels (Fig 6C), and tissue types (Fig 6D). These results implied that the matrix tissues did not exhibit such BC-dependent difference in forward scattering, since one does not expect a significant presence of BCs in the tissue outside of the blood vessels. The signals from background tissue could not be practically influenced by the localization of BCs in blood vessels as well as the administration of EB dye.

### Observation of brain hemorrhage by SF-CARS

**Fig 7:**
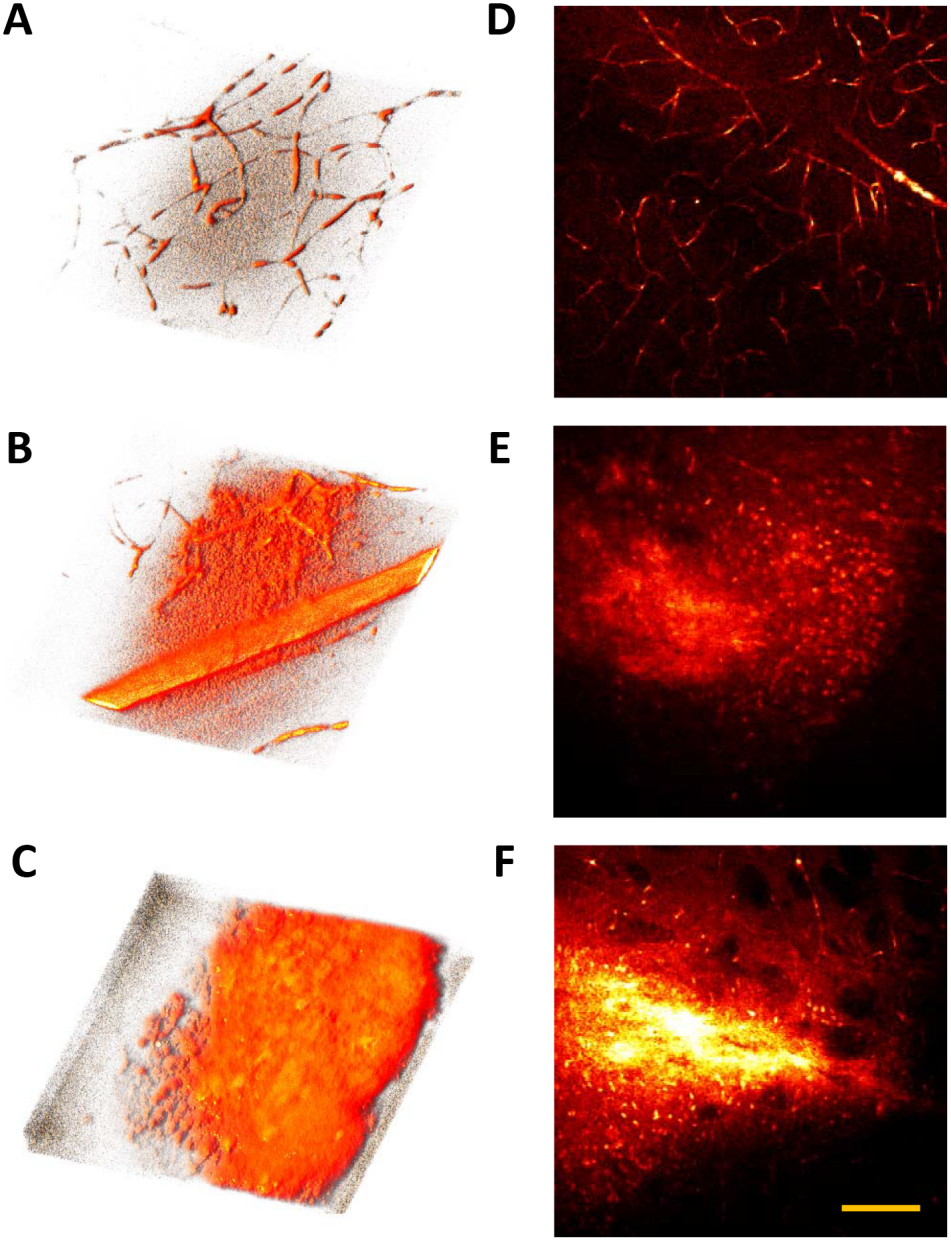
Observation of brain hemorrhage by SF-CARS. Three dimensional backward SF-CARS images of mouse brain hemorrhage induced by injection of collagenase to brain striatum for (A) and (D) 0, (B) and (E) 3 and (C) and (F) 24 hours after injection. (A)–(C) Backward SF-CARS images were taken at 2920 cm^-1^ and three dimensionality was achieved by Image J software. (D)–(F) Merged images by stacking images captured every 1 μm. The scale bar indicates 50 μm.

Finally, the potential of the use of enhanced SF-CARS signals is shown by the fact that damage of brain microvessels induced by hemorrhage could be visualized by this method. Intracerebral hemorrhage was induced by 0.5 U of bacterial collagenase dissolved in 2 μL PBS injected into the striatum of the mouse brain for 1 min. After 0 h, 3 h, and 24 h, the brain was dissected, fixed, and sectioned at a 200-μm depth, as described above. As shown in Fig 7A–7C, the damaged vasculatures were clearly observed at 3 h, showing that the continuity of vasculatures disappeared and broken vessels appeared. The increased contrast at the point of the hemorrhagic vasculature was attributed to the released blood cells accompanying the EB dye. At 24 h, damage of the vasculature was accelerated, and a dramatically increased contrast of SF-CARS signals from the released blood cells and EB dye was observed in the hemorrhage. The highly scattering nature at the 150 μm position from the glass induced by the structural hemorrhage is shown in Fig 7D–7F with the epi-CARS images.

To explain our observations, we considered the mechanism for the enhancement of contrasting effects by epi-detection: it was attributed to increments in the numbers of epi-scattering small particles (< 1 μm in diameter) [35]. The efficiency of forward CARS would originate from intact blood cells (~10 μm) and heme groups in red blood cells. In both experimental and Monte-Carlo simulation results, back-scattered signal was gradually increased until 200 μm for mouse ear (*in vivo*) [36]. Based on this report, our results on the contrasting effects by epi-detection of SF-CARS could be validated and well-justified.

In this study, EB dye was a good contrasting agent to intensify the signals of SF-CARS from the blood vessels, which could discriminate the brain microvessels from the surrounding cells, lipids, and proteins even in the presence of highly scattering media. The diffusion dynamics of EB dye leaving the blood stream can be observed by the naked eye due to its blue color, which has been used to show the hemorrhagic status of the brain microvasculature [26]. With the help of EB dye, SF-CARS microscopy can provide high-resolution microvessel images that can be used to determine whether a blood vessel network is continuous.

Several previous methods have been reported for observing brain vasculatures, such as confocal microscopy [7–9] and photoacoustic microscopy [10], which have their own characteristics: single or two photon confocal microscopy can usually provide high resolution images, and optical coherent tomography (OCT) or photoacoustic microscopy have the capability to observe target sites *in vivo*. In comparison, the SF-CARS used in this study could be adopted to visualize the signals specific for a chemical structure from a target tissue along with similar advantages provided by two photon confocal microscopy. Even though SF-CARS might be limitedly efficient in its resolution compared with several super-resolution microscopy modalities such as photoactivated localization microscopy (PALM) and stimulated emission depletion (STED) microscopy or limitedly efficient in the *in vivo*-imaging availability offered by OCT or photoacoustic microscopy, it seems likely that it can solidly visualize the distribution of biomolecules or tracers in adjacent target tissues in the presence of chemical information.

## Conclusion

Imaging of the brain microvessel is important for understanding the integrity of the BBB during the development of neurodegeneration. We report a new method to visualize the brain microvessels at a single-capillary level in mice using SF-CARS. In this study, we demonstrated EB-enhanced imaging of brain hemorrhages in mice by using epi-detection of SF-CARS microscopy. In the spectral analysis, the forward SF-CARS signals reflecting the C-H/O-H stretch were enhanced by blood cells localized in the brain microvessels. We propose the use of EB dye as a contrast agent in visualizing the brain microvessels with C-H-rich blood cells. This provides a novel platform for observation of the blood vessel integrity in brain tissue even with a highly-scattering translucent environment.

## Supporting information

S1 Fig Home-built spectral-focusing coherent anti-Stokes Raman scattering (SF-CARS) microscope. A schematic diagram for SF-CARS microscope used in this study. HW, half wave plate; P, Glen-Thomson polarizer;; TS, motorized translational stage; M, silver mirror; DM, dichroic mirror; CG, chirping glass (12 cm × 2, SF57); LS, laser scanning system; OL, objective lens; C, condenser; BF1, BF2, bandpass filters; PMT1, PMT2, photomultiplier tubes; CCD, charge coupled device combined with spectrometer.
(PDF)

S2 Fig Raman scattering and SF-CARS. The comparison between spontaneous Raman scattering (black) and SF-CARS (blue) of the dimethylsulfoxide (DMSO) solution. Inset shows the conversion ratio of Raman shift with regards to the pump-Stokes delay.
(PDF)

S3 Fig Structure and optical property of Evans blue dye. (a) Chemical structure of Evans blue dye. (b) FTIR (red) and Raman (blue) spectrum for Evans blue dye powder. Pale blue area (2850~3100 cm^-1^) corresponds to the vibrationally-interested region in this work.
(PDF)

S4 Fig Structure and optical property of hemin. (a) Chemical structure of hemin. (b) FTIR (red) and Raman (blue) spectrum for hemin powder. Pale blue area (2850~3100 cm^-1^) corresponds to the vibrationally-interested region in this work.
(PDF)
