# Supplementary material for: Evans blue dye-enhanced imaging of the brain microvessels using spectral focusing coherent anti-Stokes Raman scattering microscopy
Source: PLoS One. 2017 Oct 19;12(10):e0185519. doi: 10.1371/journal.pone.0185519 (PMC5648124; doi:10.1371/journal.pone.0185519)
Supplement: S1 Fig — A schematic diagram for SF-CARS microscope used in this study. HW, half wave plate; P, Glen-Thomson polarizer;; TS, motorized translational stage; M, silver mirror; DM, dichroic mirror; CG, chirping glass (12 cm × 2, SF57); LS, laser scanning system; OL, objective lens; C, condenser; BF1, BF2, bandpass filters; PMT1, PMT2, photomultiplier tubes; CCD, charge coupled device combined with spectrometer. (PDF) [file pone.0185519.s001.pdf]

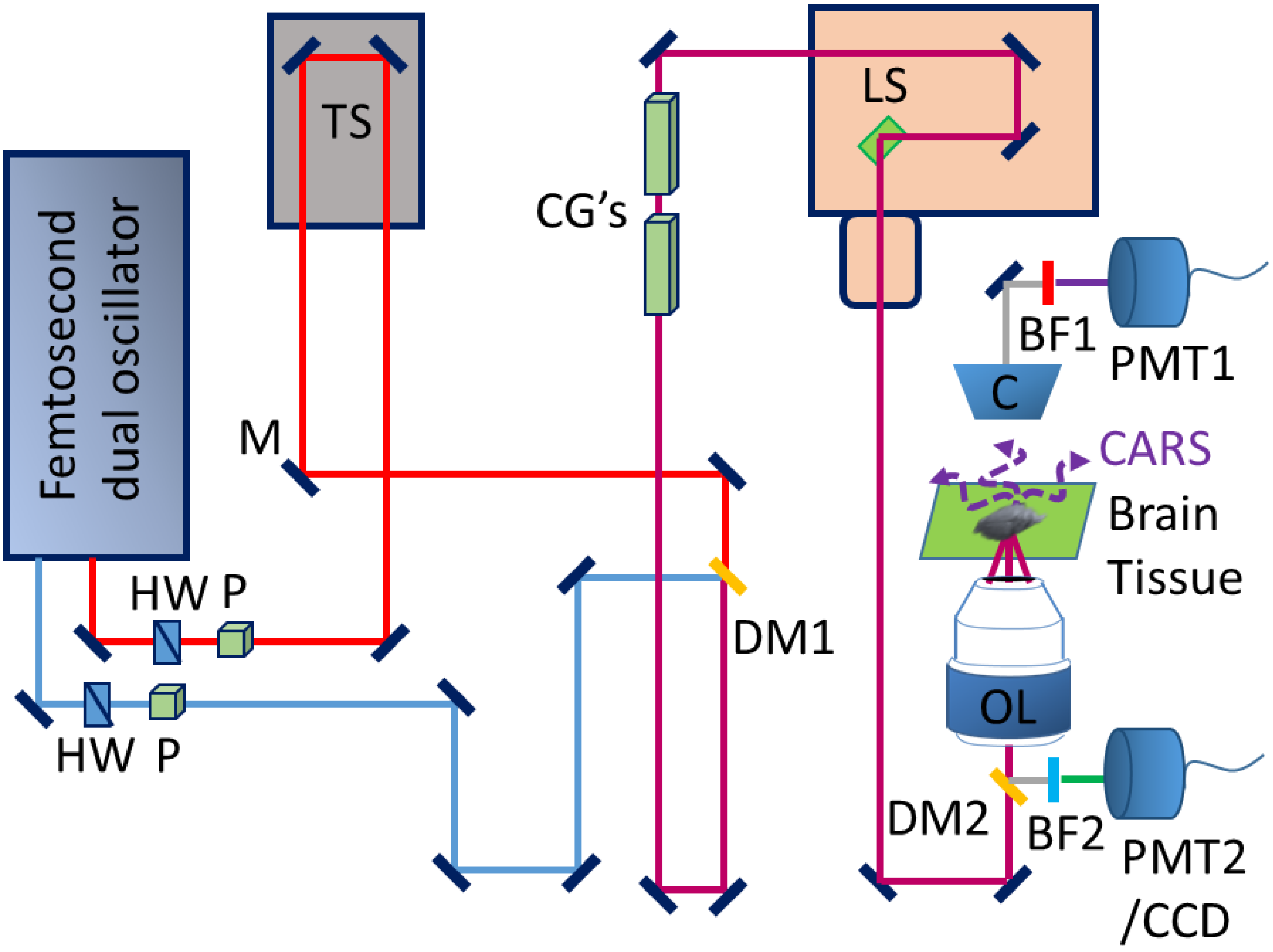

S1 Fig. Home-built spectral-focusing coherent anti-Stokes Raman scattering (SF-CARS) microscope. A schematic diagram for SF-CARS microscope used in this study. HW, half wave plate; P, Glen-Thomson polarizer;; TS, motorized translational stage; M, silver mirror; DM, dichroic mirror; CG, chirping glass (12 cm × 2, SF57); LS, laser scanning system; OL, objective lens; C, condenser; BF1, BF2, bandpass filters; PMT1, PMT2, photomultiplier tubes; CCD, charge coupled device combined with spectrometer.
