# Supplementary material for: Evans blue dye-enhanced imaging of the brain microvessels using spectral focusing coherent anti-Stokes Raman scattering microscopy
Source: PLoS One. 2017 Oct 19;12(10):e0185519. doi: 10.1371/journal.pone.0185519 (PMC5648124; doi:10.1371/journal.pone.0185519)
Supplement: S2 Fig — The comparison between spontaneous Raman scattering (black) and SF-CARS (blue) of the dimethylsulfoxide (DMSO) solution. Inset shows the conversion ratio of Raman shift with regards to the pump-Stokes delay. (PDF) [file pone.0185519.s002.pdf]

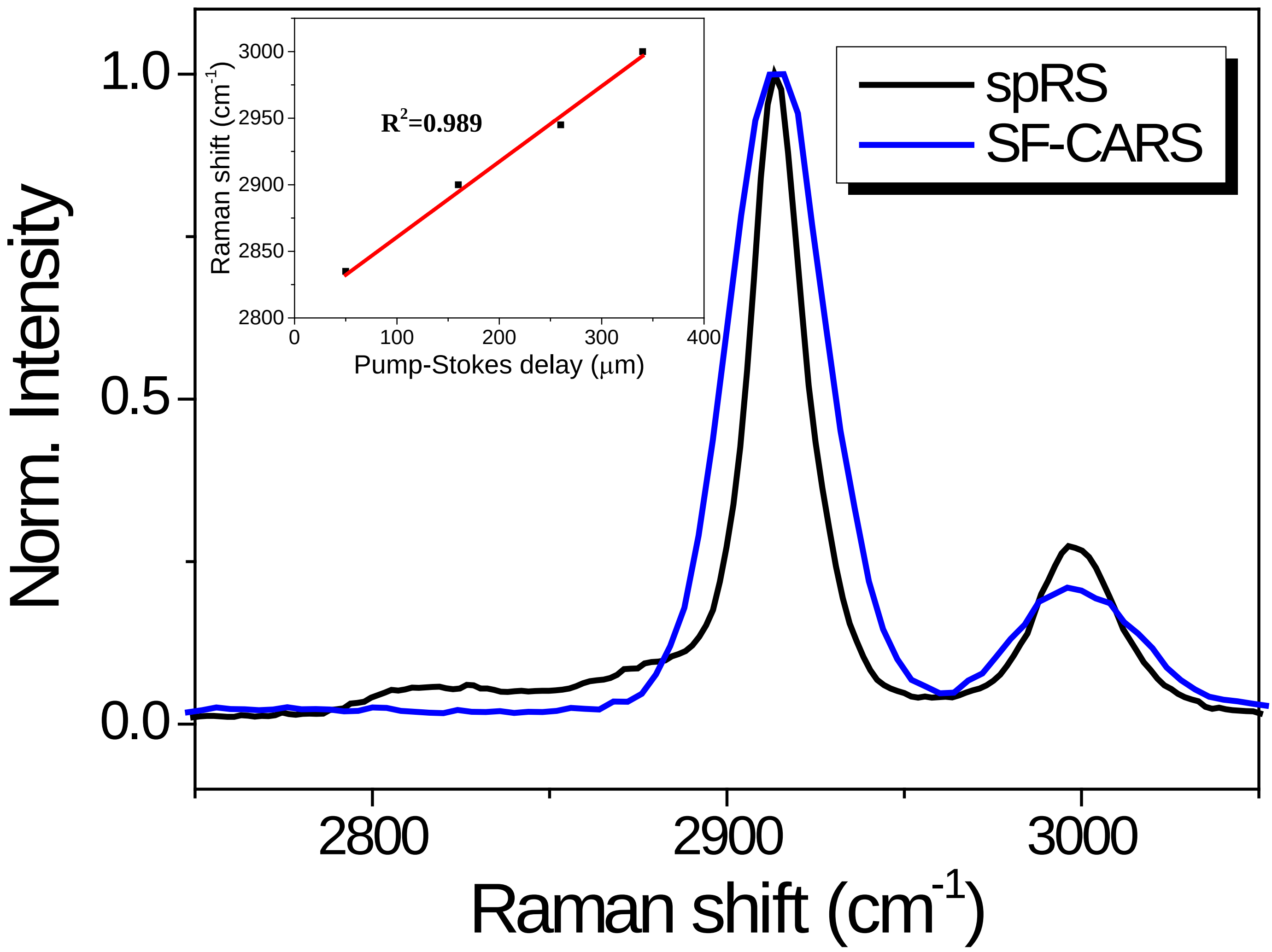

S2 Fig. Raman scattering and SF-CARS. The comparison between spontaneous Raman scattering (black) and SF-CARS (blue) of the dimethylsulfoxide (DMSO) solution. Inset shows the conversion ratio of Raman shift with regards to the pump-Stokes delay.
