# Supplementary material for: Evans blue dye-enhanced imaging of the brain microvessels using spectral focusing coherent anti-Stokes Raman scattering microscopy
Source: PLoS One. 2017 Oct 19;12(10):e0185519. doi: 10.1371/journal.pone.0185519 (PMC5648124; doi:10.1371/journal.pone.0185519)
Supplement: S3 Fig — (a) Chemical structure of Evans blue dye. (b) FTIR (red) and Raman (blue) spectrum for Evans blue dye powder. Pale blue area (2850~3100 cm-1) corresponds to the vibrationally-interested region in this work. (PDF) [file pone.0185519.s003.pdf]

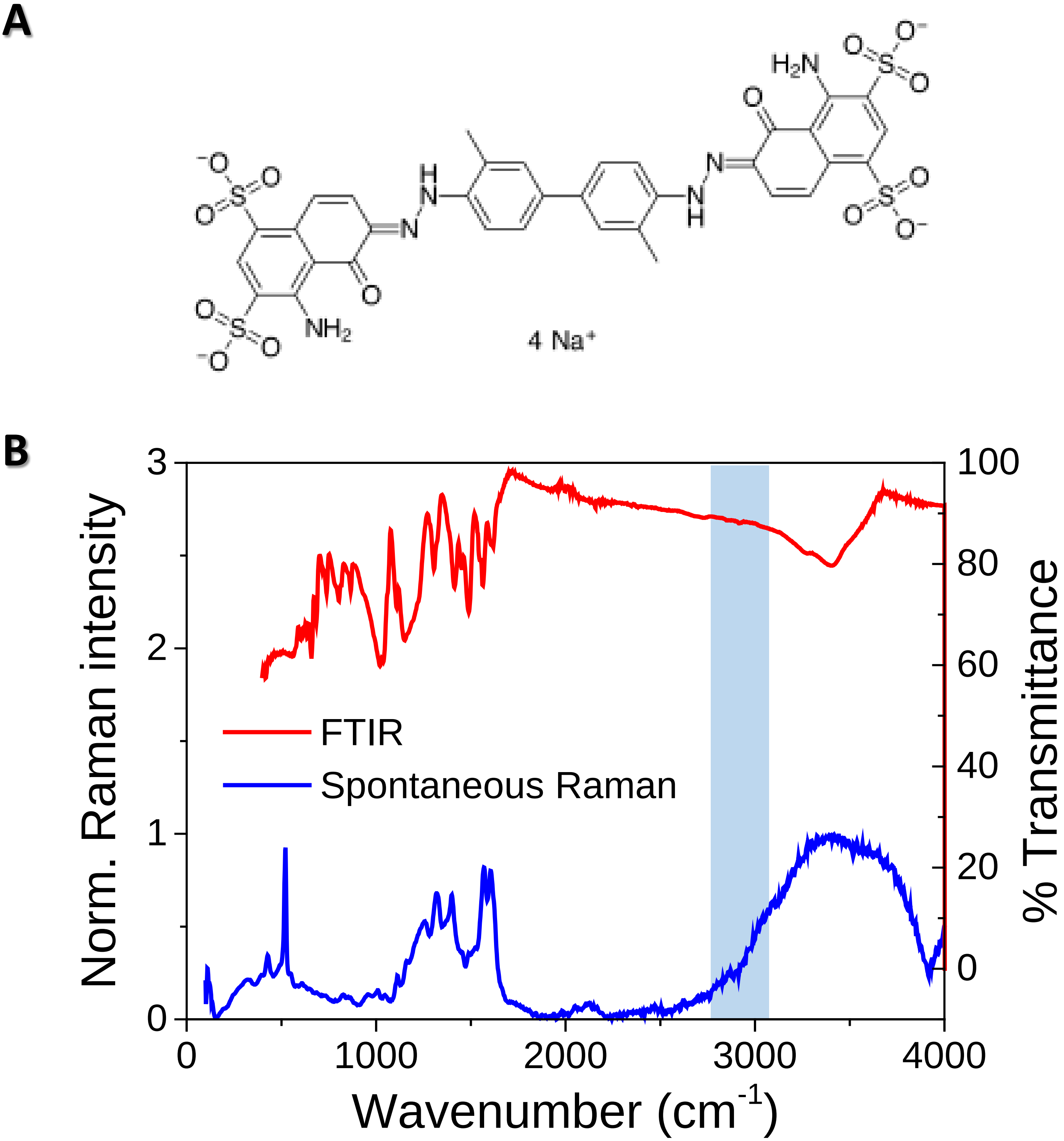

S3 Fig. Structure and optical property of Evans blue dye. (a) Chemical structure of Evans blue dye. (b) FTIR (red) and Raman (blue) spectrum for Evans blue dye powder. Pale blue area (2850~3100 cm^{-1}) corresponds to the vibrationally-interested region in this work.
