# Supplementary material for: Evans blue dye-enhanced imaging of the brain microvessels using spectral focusing coherent anti-Stokes Raman scattering microscopy
Source: PLoS One. 2017 Oct 19;12(10):e0185519. doi: 10.1371/journal.pone.0185519 (PMC5648124; doi:10.1371/journal.pone.0185519)
Supplement: S4 Fig — (a) Chemical structure of hemin. (b) FTIR (red) and Raman (blue) spectrum for hemin powder. Pale blue area (2850~3100 cm-1) corresponds to the vibrationally-interested region in this work. (PDF) [file pone.0185519.s004.pdf]

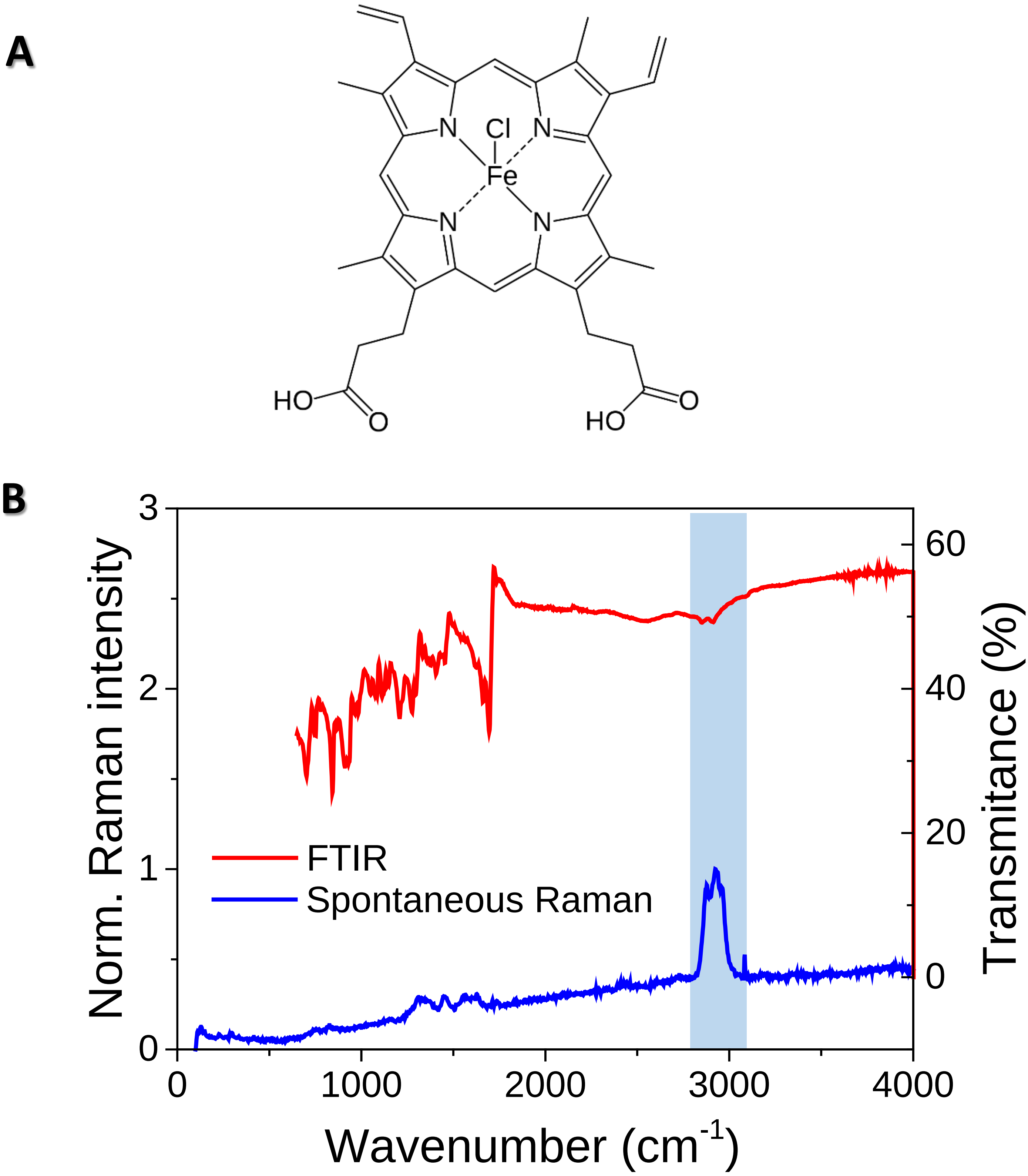

S4 Fig. Structure and optical property of hemin. (a) Chemical structure of hemin. (b) FTIR (red) and Raman (blue) spectrum for hemin powder. Pale blue area (2850~3100 cm^{-1}) corresponds to the vibrationally-interested region in this work.
